# Shaping the future of bananas: advancing genetic trait regulation and breeding in the postgenomics era

**DOI:** 10.1093/hr/uhaf044

**Published:** 2025-02-12

**Authors:** Hongxia Miao, Jianbin Zhang, Yunke Zheng, Caihong Jia, Yulin Hu, Jingyi Wang, Jing Zhang, Peiguang Sun, Zhiqiang Jin, Yongfeng Zhou, Sijun Zheng, Wei Wang, Mathieu Rouard, Jianghui Xie, Juhua Liu

**Affiliations:** National key Laboratory of Tropical Crop Breeding, Institute of Tropical Bioscience and Biotechnology & Sanya Research Institute, Chinese Academy of Tropical Agricultural Sciences, Xueyuan Road 4, Longhua District, Sanya/Haikou 571101, China; Hainan Key Laboratory for Protection and Utilization of Tropical Bioresources, Hainan Institute for Tropical Agricultural Resources, Chinese Academy of Tropical Agricultural Sciences, Xueyuan Road 4, Longhua District, Haikou 571101, China; National key Laboratory of Tropical Crop Breeding, Institute of Tropical Bioscience and Biotechnology & Sanya Research Institute, Chinese Academy of Tropical Agricultural Sciences, Xueyuan Road 4, Longhua District, Sanya/Haikou 571101, China; Hainan Key Laboratory for Protection and Utilization of Tropical Bioresources, Hainan Institute for Tropical Agricultural Resources, Chinese Academy of Tropical Agricultural Sciences, Xueyuan Road 4, Longhua District, Haikou 571101, China; National key Laboratory of Tropical Crop Breeding, Institute of Tropical Bioscience and Biotechnology & Sanya Research Institute, Chinese Academy of Tropical Agricultural Sciences, Xueyuan Road 4, Longhua District, Sanya/Haikou 571101, China; Hainan Key Laboratory for Protection and Utilization of Tropical Bioresources, Hainan Institute for Tropical Agricultural Resources, Chinese Academy of Tropical Agricultural Sciences, Xueyuan Road 4, Longhua District, Haikou 571101, China; National key Laboratory of Tropical Crop Breeding, Institute of Tropical Bioscience and Biotechnology & Sanya Research Institute, Chinese Academy of Tropical Agricultural Sciences, Xueyuan Road 4, Longhua District, Sanya/Haikou 571101, China; Hainan Key Laboratory for Protection and Utilization of Tropical Bioresources, Hainan Institute for Tropical Agricultural Resources, Chinese Academy of Tropical Agricultural Sciences, Xueyuan Road 4, Longhua District, Haikou 571101, China; Key Laboratory of Tropical Fruit Biology of Ministry of Agriculture and Rural Affairs, Key Laboratory of Hainan Province for Postharvest Physiology and Technology of Tropical Horticultural Products, South Subtropical Crop Research Institute, Chinese Academy of Tropical Agricultural Sciences, Xiuhu Road 1, Mazhang District, Zhanjiang 524000, China; National key Laboratory of Tropical Crop Breeding, Institute of Tropical Bioscience and Biotechnology & Sanya Research Institute, Chinese Academy of Tropical Agricultural Sciences, Xueyuan Road 4, Longhua District, Sanya/Haikou 571101, China; Hainan Key Laboratory for Protection and Utilization of Tropical Bioresources, Hainan Institute for Tropical Agricultural Resources, Chinese Academy of Tropical Agricultural Sciences, Xueyuan Road 4, Longhua District, Haikou 571101, China; National key Laboratory of Tropical Crop Breeding, Institute of Tropical Bioscience and Biotechnology & Sanya Research Institute, Chinese Academy of Tropical Agricultural Sciences, Xueyuan Road 4, Longhua District, Sanya/Haikou 571101, China; Hainan Key Laboratory for Protection and Utilization of Tropical Bioresources, Hainan Institute for Tropical Agricultural Resources, Chinese Academy of Tropical Agricultural Sciences, Xueyuan Road 4, Longhua District, Haikou 571101, China; National key Laboratory of Tropical Crop Breeding, Institute of Tropical Bioscience and Biotechnology & Sanya Research Institute, Chinese Academy of Tropical Agricultural Sciences, Xueyuan Road 4, Longhua District, Sanya/Haikou 571101, China; Hainan Key Laboratory for Protection and Utilization of Tropical Bioresources, Hainan Institute for Tropical Agricultural Resources, Chinese Academy of Tropical Agricultural Sciences, Xueyuan Road 4, Longhua District, Haikou 571101, China; National key Laboratory of Tropical Crop Breeding, Institute of Tropical Bioscience and Biotechnology & Sanya Research Institute, Chinese Academy of Tropical Agricultural Sciences, Xueyuan Road 4, Longhua District, Sanya/Haikou 571101, China; National key Laboratory of Tropical Crop Breeding, Institute of Tropical Bioscience and Biotechnology & Sanya Research Institute, Chinese Academy of Tropical Agricultural Sciences, Xueyuan Road 4, Longhua District, Sanya/Haikou 571101, China; National Key Laboratory of Tropical Crop Breeding, Shenzhen Branch, Guangdong Laboratory of Lingnan Modern Agriculture, Key Laboratory of Synthetic Biology, Ministry of Agriculture and Rural Affairs, Agricultural Genomics Institute at Shenzhen, Chinese Academy of Agricultural Sciences, Pengfei Road 7, Dapengxin District, Shenzhen 518000, China; Yunnan Key Laboratory of Green Prevention and Control of Agricultural Transboundary Pests, Agricultural Environment and Resources Institute, Yunnan Academy of Agricultural Sciences, Beijing Road 2238, Kunming 650205, China; Bioversity International, Yunnan Academy of Agricultural Sciences, Beijing Road 2238, Kunming 650205, China; National key Laboratory of Tropical Crop Breeding, Institute of Tropical Bioscience and Biotechnology & Sanya Research Institute, Chinese Academy of Tropical Agricultural Sciences, Xueyuan Road 4, Longhua District, Sanya/Haikou 571101, China; Hainan Key Laboratory for Protection and Utilization of Tropical Bioresources, Hainan Institute for Tropical Agricultural Resources, Chinese Academy of Tropical Agricultural Sciences, Xueyuan Road 4, Longhua District, Haikou 571101, China; Bioversity International, Parc Scientifique Agropolis II, Montpellier 34397, Cedex 5, France; National key Laboratory of Tropical Crop Breeding, Institute of Tropical Bioscience and Biotechnology & Sanya Research Institute, Chinese Academy of Tropical Agricultural Sciences, Xueyuan Road 4, Longhua District, Sanya/Haikou 571101, China; National key Laboratory of Tropical Crop Breeding, Institute of Tropical Bioscience and Biotechnology & Sanya Research Institute, Chinese Academy of Tropical Agricultural Sciences, Xueyuan Road 4, Longhua District, Sanya/Haikou 571101, China; Hainan Key Laboratory for Protection and Utilization of Tropical Bioresources, Hainan Institute for Tropical Agricultural Resources, Chinese Academy of Tropical Agricultural Sciences, Xueyuan Road 4, Longhua District, Haikou 571101, China

## Abstract

Bananas (*Musa* spp.) are among the top-produced food crops, serving as a primary source of food for millions of people. Cultivated bananas originated primarily from the wild diploid species *Musa acuminata* (A genome) and *Musa balbisiana* (B genome) through intra- and interspecific hybridization and selections via somatic variation. Following the publication of complete A- and B-genome sequences, prospects for complementary studies on S- and T-genome traits, key gene identification for yield, ripening, quality, and stress resistance, and advances in molecular breeding have significantly expanded. In this review, latest research progress on banana A, B, S, and T genomes is briefly summarized, highlighting key advances in banana cytoplasmic inheritance, flower and fruit development, sterility, and parthenocarpy, postharvest ripening and quality regulation, and biotic and abiotic stress resistance associated with desirable economic traits. We provide updates on transgenic, gene editing, and molecular breeding. We also explore future directions for banana breeding and genetic improvement.

## Introduction

Bananas rank as one of the top food crops and serve as a staple food for millions. This fruit holds major economic value, accounting for over 16% of global production and trade in fresh produce [1–3]. Botanically, bananas belong to the Musaceae family and are classified as monocotyledonous plants within the *Musa* genus*.* Species in the genus were initially divided into five sections, namely *Eumusa* (2*n* = 22), *Australimusa* (2*n* = 20), *Rhodochlamys* (2*n* = 22), *Callimusa* (2*n* = 18 or 20), and *Ingentimusa* (2*n* = 14) [4]; later, they have been subdivided into two sections, namely *Musa* (*Eumusa* and *Rhodochlamys*) and *Callimusa* (*Australimusa, Callimusa*, and *Ingentimusa*), based on phylogenetic analyses [5, 6]. More than 70 wild species exist in the *Musa* genus, typically distinguished by their gravelly hard seeds and minimal pulp [5]. The evolution and selection from wild to edible bananas involved seedlessness, improved plant vigour, increased hardiness, and higher yield [7]. For cultivated bananas, estimates of diversity range from 500 to over 1000 cultivars, encompassing various genomic combinations [4, 8], and comprise various genomic combinations, including AA, BB, AB, AAA, AAB, ABB, and less frequent hybrids like AS, AT, AAS, AAT, AAAT, AAAB, AABB, ABBB, and ABBT [9–12]. They have mainly resulted from hybridization within *M. acuminata* subspecies (A genome, 2*n* = 22) and *Musa balbisiana* (B genome, 2*n* = 22). *M. schizocarpa* (S genome, 2*n* = 22) and species belonging to the *Australimusa* section, *Musa textilis* (T genome, 2*n* = 20) [7, 10–12]. As bananas were exchanged across countries, their diversity has been conserved through the management and documentation of thousands of accessions in more than 30 *ex situ* collections worldwide [9].

Bananas are a perfect genetic model for investigating bi-parental cytoplasmic inheritance, plant evolution, and co-evolution of plants and pathogens [7, 10]. Banana serves also as a model for respiratory climacteric fruits and starch conversion, requiring autocatalytic ethylene for ripening and starch breakdown [13, 14]. During ripening, bananas undergo a series of complex changes that simultaneously affect fruit quality, including alterations in colour, texture, flavour, aroma, and secondary metabolite content [15]. Most cultivated bananas are triploid, with large-scale vegetative propagation. The genetic restrictions of sterility and parthenocarpy make traditional breeding difficult and time-consuming. Therefore, modern molecular breeding technologies have become an inevitable requirement of banana genetic improvement and new variety breeding.

In 2012, the first complete reference genome for *M. acuminata* ssp. *malaccensis* (A genome, DH-Pahang) was unveiled [16], marking it a groundbreaking milestone. Seven years later, the release of the reference B genome sequence for *M. balbisiana* (DH-PKW) [17] further enriched our understanding, shedding light on the functional diversification of subgenomes. Given the relevance of knowledge for advancing banana crop improvement, there has been a growing focus on sequencing and re-sequencing the genomes of wild species (S and T-genome) and related cultivars [3, 18–22]. Recently, the origin and evolution of the triploid cultivated banana genomes (AAA and AAB) were reported [21, 22]. The increased quantity of sequencing data has enabled the creation of freely accessible resources for functional genomics, leading to the development of the Banana Genome Hub, a portal that supports the analysis and understanding of genetic mechanisms underlying agronomic traits [23].

This review presents an overview of the genetic regulation of important traits in bananas and highlights their utilization for genetic improvement. It elaborates on recent advancements in multidimensional omics for an in-depth exploration of candidate genes, highlighting their potential in developing improved banana varieties through conventional breeding or genetic engineering. Unravelling the genetic mechanisms behind key traits holds great potential for boosting banana germplasm improvement in the postgenomics era.

## Banana genome sequences

The genome sequencing strategy has initially focused on wild relatives of cultivated bananas, particularly diploids using double haploids to simplify the genome assembly process [16, 17]. As sequencing technologies have advanced, more complete genomes and higher levels of heterozygosity have been achieved, leading to more complex genomes for cultivated bananas due to their polyploidy [10, 11]. Further details are provided below.

The first A genome sequence of banana cultivar ‘DH-Pahang’ (*M. acuminata*) has been completed through Sanger and Illumina short read (SR) technology [16]. Originally, a total of 7513 scaffolds have been assembled for the A genome, with an N50 length of 1.3 Mb, covering 90% (472.2 Mb) of the estimated 523 Mb genome (Table 1). Using a highly saturated genetic map, 332 Mb sequences, representing 70% of the assembled genome, have been mapped on all 11 chromosomes of the banana A genome. Repeats sequences accounted for 41.85% of the assembled genome. In addition, 36 542 protein-coding genes and 235 microRNAs from 37 families have been identified. Nearly 10 years later, this sequence was refined through two additional iterations, resulting in an almost gapless version where complex regions are better captured, and the size of chromosomes has increased [24]. Additional *M. acuminata* subspecies were also sequenced and assembled as draft genomes to better characterize the diversity of the A genome found in cultivated bananas [25]. These assemblies are currently being refined and improved to high-quality standards.

**Table 1:** Comparisons among published wild and cultivated banana genomes

Species/cultivated group	Genome	Genotype name	Technology	Comments	Genome size	N50 length (Mb)	Coding genes	References
*Musa acuminata*	A	DH Pahang	Sanger, Illumina SR	First version	523 Mb	1.3	36 542	[16]
*Musa balbisiana*	B	DH Pisang Klutuk Wulung (PKW)	Illumina SR, PacBio LR, Hi-C	First version	430 Mb	5.05	35 148	[17]
*Musa schizocarpa*	*S*	Schizocarpa	Nanopore LR, Bionano	First version	505 Mb	3.68	32 809	[19]
*Musa textilis*	T	Abaca	Illumina NovaSeq 6000	First version	613 Mb	3.5	35 077	[6]
*Musa troglodytarum*	TT	Utafun	Nanopore LR, Illumina SR, PacBio LR, Hi-C	First version	603 Mb	4.9	37 577	[20]
*M. troglodytarum*	TTT	Karat	Nanopore LR, Illumina SR, PacBio LR, Hi-C	First version	619 Mb	3.6	36 080	[20]
*M. acuminata*	AAA	Cavendish	PacBio LR, Illumina SR, Hi-C	First version	1.48 Gb	0.24	106 540	[22]
*M. acuminata*	AAA	Gros Michel	PacBio LR, Illumina SR, Hi-C	First version	1.33 Gb	1.04	120.653	[22]
*M.* spp.	AAB	Plantain (French Horn)	Illumina SR, PacBio LR, Hi-C	First version	1.69 Gb	2.01	88 078	[21]
*M.* spp.	AAB	Silk (Figue Pomme Géane)	Illumina SR, PacBio LR, Hi-C	First version	1.52 Gb	2.92	94 988	[21]

Compared with the A genome, the B genome sequence is a chromosome-scale assembly from the banana cultivar ‘DH-PKW’ (*M. balbisiana*) completed using Illumina SR, PacBio long read (LR), and high-throughput chromosome conformation capture (Hi-C) technology [17]. A combined 58.99 Gb (113×) of PacBio long-read sequences and 86.34 Gb (166×) of Illumina short-read data, including both paired-end and mate-pair reads, have been assembled for the B genome with an N50 of 5.05 Mb, resulting in 492.77 Mb of scaffolds (Table 1). Based on a Hi-C library of DH-PKW, approximately 87% of the assembly, totalling 430 Mb, and 94% of genes have been assigned to the 11 chromosomes. The B-genome assembly consists of 55.75% repetitive sequences, exceeding the 41.85% found in the A-genome assembly. In addition, 35 148 protein-coding genes and 3329 transcription factors (TFs) have been identified for this assembly.

The S genome sequence, as a chromosome-scale assembly of banana (*M. schizocarpa*), has been completed using Nanopore LR technology [19]. A total of 227 scaffolds (≥2 kb) have been assembled for the banana S genome with an N50 of 3.68 Mb, representing 92.3% of the estimated genome size (587 Mb) (Table 1). A total of 505 Mb of sequences, accounting for 86% of the S genome assembly, have been placed on all 11 chromosomes, with further validation from the genetic map. Repetitive sequences made up 47% of the assembled genome, with 32 809 protein-coding genes identified. The whole-genome comparison between *M. schizocarpa* and *M. acuminata* revealed significant variability in their centromeric regions.

The T genome sequence of banana accessions ‘Karat’ and ‘Utafun’ (*Musa troglodytarum*, Fe’i group), ‘Abaca’ (*M. textilis*) has been completed using Nanopore LR, Illumina SR, PacBio LR, and Hi-C technology [6, 20]. For ‘Karat’, 42 Gb of Nanopore reads, 6.9 Gb of PacBio reads, and 42 Gb of Illumina reads have been assembled with an N50 of 4.9 Mb (Table 1). Following the removal of haplotigs, 603 Mb of contigs and 110 Gb of Hi-C reads were assembled into 10 chromosomes of the banana T genome, achieving a BUSCO score of 97.7% for gene completeness and including 59.62% of repeat sequences. For the ‘Karat’, ‘Utafun’, and ‘Abaca’ accessions, 37 577, 36 080, and 35 077 protein-coding genes have been identified, respectively.

The AAA genome sequences of banana accessions ‘Cavendish’ (*M. acuminata* cv. Cavendish) and ‘Gros Michel’ (*M. acuminata* cv. Gros Michel) have been completed using PacBio sequencing, Illumina sequencing, and Hi-C technologies [22]. The ‘Cavendish’ and ‘Gros Michel’ genome assemblies possess 6765 contigs (N50 = 0.24 Mb) and 6423 contigs (N50 = 1.04 Mb) spanning 1.48 Gb and 1.33 Gb, respectively (Table 1). Using Hi-C data, 1.23 Gb (83.4%) of ‘Cavendish’ and 1.32 Gb (99.1%) of ‘Gros Michel’ contig sequences were anchored onto 33 chromosomes of the banana AAA genome, achieving the BUSCO score of 97.7% (‘Cavendish’) and 96.9% (‘Gros Michel’) for gene completeness. For the ‘Cavendish’ and ‘Gros Michel’ accessions, 106 540 and 120 653 protein-coding genes have been identified, respectively.

The AAB genome sequences of banana accessions Plantain (*M.*  spp. cv. French Horn) and Silk (*M.* spp. cv. Figue Pomme Géane) have been completed using Illumina SR, PacBio LR, and Hi-C technologies [21]. The Plantain and Silk genome assemblies generated with contig N50 values of 2.01–2.92 Mb spanning 1.69 and 1.52 Gb, respectively (Table 1). Using Hi-C data, over 90% of the Plantain reads and 93% of the Silk reads were anchored to the final 33 chromosomes of the banana AAB genome, achieving an average of ~92% BUSCO plant reference genes in each assembly. For the Plantain and Silk accessions, 88 078 and 94 988 protein-coding genes have been identified, respectively.

Leveraging reference genomes and resequencing data, it has become possible to uncover homoeologous recombination that exists between A and B genomes [26, 27]. Furthermore, reciprocal translocations between them have been observed. Notably, chromosomal translocations involving chromosomes 1 and 3, along with an inversed segment on chromosome 5, have been documented [17, 26]. Additional translocations within the A genome have also been identified and can affect chromosome segregation and potentially explain complications in breeding efforts [10].

Given the nature of the crop, researchers have been able to explore the ancestral contributions to cultivated bananas through *in silico* chromosome painting, which has clarified the domestication process of bananas [10, 11]. Based on this approach, a chromosome-based catalog of cultivated bananas, clarifying the ancestral contributions of most of the defined cultivar groups, was released [28]. Some regions of the genome have been traced back to different origins, with some regions potentially linked to traits of interest that could be leveraged in breeding programs [29].

As more genomes become available, the development of pangenomes offers an expanded view of the genomic diversity of bananas. The first pangenome, constructed with draft genomes representing around 15 different *Musa* and Enset genomes, highlighted significant gene presence-absence variation [30]. The shift towards more comprehensive telomere-to-telomere genome assemblies in bananas is expected to bring new type of pangenomes that will enhance our understanding of structural variations, leading to more informed strategies for crop improvement [31, 32]. All these elements represent crucial genomic insights that can be leveraged to develop more targeted breeding strategies.

## Cytoplasmic inheritance

Traits inherited through the cytoplasm, including resistance to viruses and fungi, regulation of sugar synthesis, starch storage, production of certain amino acids, lipids, pigments, and vitamins, as well as crop yield, temperature tolerance, and essential nitrogen and sulphur metabolic pathways, frequently play a role in beneficial phenotypic characteristics in plants [10].

The plastomes of the Musaceae family exhibit a well-conserved genomic size and gene content, averaging approximately 169 648 bp [33]. Fauré *et al.* [34] observed a distinct pattern of maternal inheritance for chloroplast DNA (cpDNA) and paternal inheritance for mitochondrial DNA (mtDNA) in *M. acuminata*. This result suggests the presence of two distinct mechanisms for organelle transmission and selection in bananas, likely due to the prevalence of parthenocarpy and triploidy. Boonruangrod *et al.* [35] reported an in-depth analysis of the chloroplast and mitochondrial genomes in *M. acuminata* and *M. balbisiana*, identifying six distinct chloroplast gene pools and seven mitochondrial gene pools among the accessions studied. The strong prevalence of specific cytoplasmic gene pools in cultivars suggests that centuries of natural and farmer-driven selection may have complemented the phenotypic traits shaped by the nuclear genome.

The mitochondrial *NAD1* gene encoding mitochondrial nicotinamide adenine dinucleotide dehydrogenase subunit 1 has been used to phylogenetically position banana cultivars in comparison to diploid progenitors [36]. The chloroplast genome of *M. acuminata* contains 112 genes, including 79 protein-coding genes, 29 transfer RNA (tRNA) genes, and 4 ribosomal RNA (rRNA) genes. The majority of genes are present as single copies, while 23 gene types appear in duplicate [37, 38]. The *M. balbisiana* chloroplast genome encodes 113 unique genes, including 79 protein-coding genes, 30 tRNA genes, and rRNA genes. Currently, over 60 complete chloroplast genomes of banana species, along with some cultivars, are available, providing valuable insights for developing DNA markers and supporting future population genetics research and conservation efforts for bananas [39–41].

## Banana vegetative morphology and growth

*Musa* plants are among the largest herbaceous perennials and consist of underground (corms, roots, and suckers) and aboveground parts (pseudostem, leaf, inflorescence, and fruit). Banana growth is a complex process influenced by many environmental and genetic factors. Bananas with ideal plant architecture (IPA) exhibit a dwarf phenotype characterized by robust stems, more upright leaves, and a deep root system [42]. Developing crops with IPA has the potential to enhance yield [43]. Manipulating the *terpene synthase* (*TPS*), *Cytochrome P450 monooxygenases* (*P450s*), *gibberellin 20ox2* (*MaGA20ox2*), and *GA 3-oxidas*e (*GA3ox*) genes can generate a semidwarf phenotype by disrupting the GA pathway [42, 44]. The *MaTCP* gene family plays an important role in regulating banana pseudostem growth and development [45]. Banana PYL-PP2C-SnRK2-mediated abscisic acid (ABA) signalling is involved in tissue development [46]. NAC TFs play an important role in the regulation of secondary wall deposition [47]. Overexpression of *MaVND1*, *MaVND2*, and *MaVND3* results in ectopic lignin deposition in various cells [47, 48].

## Flower development

**Figure 1:**
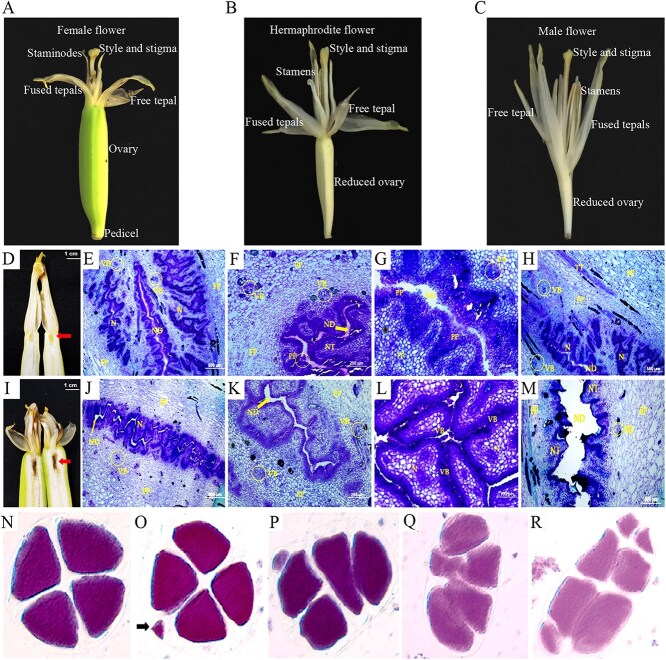
Morphoanatomical structure characteristics of banana female and male flowers. A: Female flower; B: Hermaphrodite flower; C: Male flower. D-H: Morphoanatomical characteristics of female flowers of ‘Calcutta 4’ (AA genotype); I-M: Morphoanatomical characteristics of female flowers of ‘Grande Naine’ (AAA genotype) [51]. ND, nectariferous duct; VB, vascular bundle; LO, loculus; N, nectar; FP, fundamental parenchyma; PP, pluristratified papillae; NT, nectariferous tissue; TT, transmitting tissue. N-R: tetrad analysis of male flower in *M. acuminata*. N, tetrad [52]; O, tetrad with micronucleus as arrow point; P, microspore with different shape; Q: pentad; R, heptad

Floral initiation begins when the apical meristem stops leaf production and grows through the center of the pseudostem, eventually leading to the emergence of the inflorescence bud. The first floral organ formation can be detected in 9–11 leaves. Inflorescence emergence is the process by which inflorescences shoot vertically from the pseudostem [8]. The flowering time is dependent largely on the cultivar, total expanded leaf area, and environmental conditions [49]. Inflorescences are stout, ovate spikes encased in large, spiny bracts that are usually purple-red in colour [50]. There are three types of flowers that are bisexual but mostly unisexual in function: lower bracts of inflorescences enclose female flowers with an ovary length greater than that of the stigma and style (Fig. 1A), middle few bracts enclose neutral or hermaphroditic flowers with an ovary length equal to the length of the stigma plus the style (Fig. 1B), and the top of the inflorescence encloses male flowers with an ovary length less than that of the stigma and style (Fig. 1C). The three flower types arise from fundamentally similar flower primordia, with their development primarily influenced by nutrient availability and allocation [4].

One of the major challenges in banana improvement lies in the low fertility of female flowers in most commercial cultivars, which severely limits hybridization, resulting in either very few seeds, small progeny, or a complete lack of seeds [51, 53]. The occurrence of female sterility is influenced by a range of parameters: (i) low pollen grain germination rates, (ii) slow and irregular pollen tube growth leading to nonfertilized ovules, and (iii) female flowers showing failure in embryo sac development [4, 53–55]; and (iv) recently, some studies have shown that the nectaries of female flowers undergo oxidation or necrosis, which hinders female fertility [51, 56]. In ‘Calcutta 4’ (*M. acuminata* ssp. *burmannica*, AA genotype), the female flowers were smaller (Fig. 1D) and the nectariferous ducts were narrower (Fig. 1E–G) than those in ‘Grande Naine’ (Cavendish, AAA genotype) (Fig. 1I–L). This narrowing may help guide the pollen tube more accurately and allow for smoother passage, improving the chances of ovule fertilization [51, 57]. Transmitting tissue has only been observed in ‘Calcutta 4’, running alongside the nectar zone (Fig. 1H). However, this tissue is absent in ‘Grande Naine’ (Fig. 1M), which does not exhibit pollen tube growth, prohibiting fertilization [58]. Differences in pollen tube growth between the two genotypes have also been noted at anthesis. In 'Grande Naine', fewer pollen tubes have been observed in the lower third of the style compared to the wild 'Calcutta 4', with a particularly significant reduction in the nectary zone [51]. This demonstrates that no physical barriers are preventing fertilization in diploid genotype bananas.

Cultivated banana varieties are almost male sterile and produce little to no viable pollen [59–61]. By searching the Web of Science database, only six SCI papers have been found to report the development of male flowers in bananas, and they mainly focus on microspore detection and pollen vitality [56, 61, 62]. Through observation of the tetrad stage of meiosis in the pollen mother cells of *M. acuminata*, most of the tetrads (92.1%) during this stage have been shown to consist of four uniformly sized microspores encapsulated in callus (Fig. 1N), but a certain proportion (7.9%) of polyad, heteromorphic nucleus, and micronucleus structure (Figs. 1O–R) have also been found [54], implying that the reason for the partial loss of pollen vitality in *M. acuminata* is likely due to irregular meiosis in pollen mother cells (PMCs). Moreover, in the later stage, obvious chromosome bridges and fragments can be observed [52]. These results indicate deficiencies in the cell division cycle of pollen mother cells in *M. acuminata*.

## Sterility and parthenocarpy

Parthenocarpy and female sterility were key traits selected during the domestication of bananas to produce seedless fruits. As a consequence, vegetative propagation became the primary method of reproduction to maintain these desirable traits. However, sterility and parthenocarpy are constraints for breeding efforts. Female sterility in edible diploid bananas is attributed to genetically regulated parthenocarpy. Parthenocarpy in banana is thought to be a characteristic of the A genome [4]. Parthenocarpy in bananas is hypothesized to result from the interaction of a major dominant gene (P or P1) with minor ones [4, 55]. Backiyarani *et al.* [63] suggested MaAGL8, MaMADS16, MaMADS29, MaGH3.8, MaRGA1, MaEXPA1, MaGID1C, MaBAM1, and MaHK2 as potential target genes for investigating natural parthenocarpy, while *MaACLB-2* and *MaZEP* are expected to play roles in both artificially induced and natural parthenocarpy. Moreover, the importance of the cytokinin-driven CLAVATA (CLV)-WUSHEL (WUS) signaling pathway, alongside the gibberellin-regulated auxin signaling involved in parthenocarpy, has been suggested. In the first genome-wide association study (GWAS) in banana, Sardos *et al.* [55] looked at the seedlessness phenotype (encompassing both sterility and parthenocarpy traits) and identified 13 candidate genomic regions associated with both traits. Histidine Kinase *CKI1* has been proposed as a good candidate gene for female sterility. Elucidating the precise mechanism of parthenocarpy would contribute greatly to high-quality fruit production.

## Fruit development and starch accumulation

Flower development is also a fruit growth and development process. After flowering, the rapid growth of the inflorescence stem/bunch results in a single fruit being filled with starch. While some bananas have upright bundles, the weight of the fruit typically causes the main stem or bunch to bend, allowing it to hang vertically. Fruit develops from the inner layer of the ovary wall and the locule septum and axis of the pistillate flowers [64]. Ovule degeneration in mature fruit is identifiable by small brown flecks. Fruit sterility occurs due to complicated causes such as specific female sterility genes, triploidy, and chromosome structural changes [49]. Parthenocarpic development is stimulated by autonomous auxin and GA production and a decrease in ABA levels in the mature ovary, supported by an increased expression level of correlated phytohormone metabolic genes at the early fruit growth and development stage [65]. Two MADS-box TFs, MuMADS1 and MaMADS7, are specifically and dominantly expressed in flowers and developing ovaries, playing important roles in banana flower and fruit development [66, 67]. MaGSTs are involved in various fruit development stages [68].

**Figure 2:**
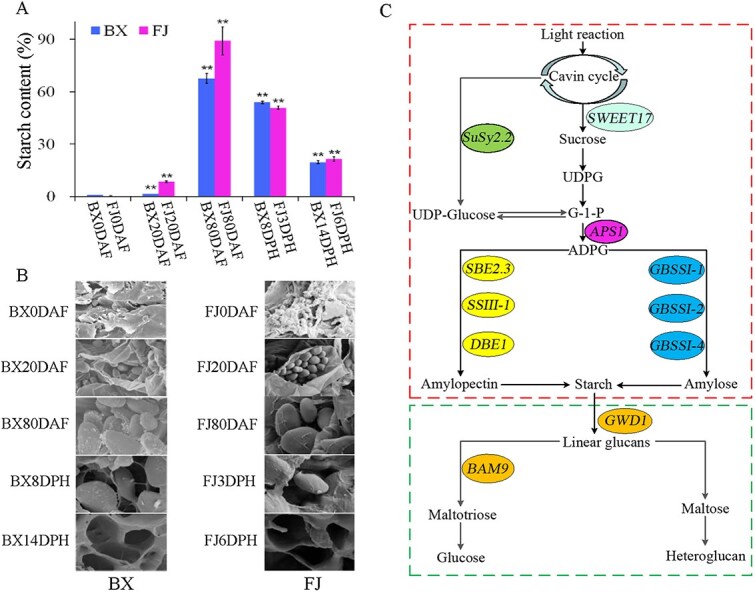
Starch synthesis metabolisms of banana fruits. A: Starch content of banana fruits. FJ, Fen Jiao (*Musa* spp. AAB, cultivar Fenjiao); BX, BaXi Jiao (*M. acuminata*, AAA, cultivar Cavendish); DAF, days after emergence stages from the pseudostem; DPH: days of postharvest. B: Changes in starch granules in banana pulp at different development stages. C: Starch synthesis pathway of banana. *Susy2.2*, *sucrose synthase gene 2.2* [72]; *SWEET17*, *sugars will eventually be exported transporter 17* [73]; *APS1*, *ADP-glucose pyrophosphorylase small subunits* [74]; *SBE2.3*, *starch branching enzyme-encoding gene 2.3* [70]; *SSIII-1*, *soluble starch synthase III-1* [75]; *DBE1*, *starch debranching enzyme 1* [17]; *GBSSI-1*, *I-2*, and *I-4*, *granule-bound starch synthase I-1*, *I-2*, and *I-4* [73]; *GWD1*, *glucan water dikinase 1* [15]; *BAM9*, *β-amylase 9* [76]

Starch is a major yield and quality component in cultivated bananas, accumulating to high levels at harvest (20%–25% of the fresh weight or 60%–75% of the dry weight) [17, 69–71]. This process is highlighted by the formation of substantial starch granules, ranging from 8 to 30 μm in size (Fig. 2A and B). Bananas provide an intriguing model for studying starch biosynthesis in freshly ripened starchy fruits. This process involves nine key enzymes: Sugars Will Eventually Be Exported Transporter (SWEET), sucrose synthase (SuSy), sucrose transporter (SUT), ADP-glucose pyrophosphorylase (AGPase), UDP-glucose pyrophosphorylase (UGPase), soluble starch synthase (SS), granule-bound starch synthase (GBSS), starch branching enzyme (SBE), and starch debranching enzyme (DBE) [70, 73–75, 77–79]. The starch production pathway has been mapped to 77 genes in the A genome and 88 genes in the B genomes, highlighting the genetic complexity underlying starch biosynthesis in bananas [16, 17]. Furthermore, five of the nine gene families—SuSy, GBSS, SS, SBE, and DBE—have shown a notable increase in the genomic diversity of A and B lineages relative to other plant species [17].

In our previous research, a diverse set of genes were found involved in starch biosynthesis throughout banana fruit development, including *MaSWEET4b*, *MaSusy2.2*, *MaAPS1*, *MaGBSSI-3*, *MaSSIII-1*, and *MaSBE2.3*, have been identified [17, 70, 72–75, 80]. A model of starch biosynthesis in banana has been constructed based on our research (Fig. 2C). In the model, *MaSWEET4b*, *MaSusy2.2*, and *MaAPS1* are mainly involved in total starch synthesis in banana fruit [72–74]. High *MaGBSSI-3* expression at transcript and protein levels is the most consistent with changes in amylose and resistant starch content in banana fruit during development [80]. *MaSSIII-1* overexpression mainly led to severe cracks in the surface of starch granules and significantly increased the total starch accumulation, amylopectin content, and SS activity in fruit [75]. Transient silencing of *MaSBE2.3* expression in banana fruit led to a significant reduction in total starch and amylopectin content [70]. Additionally, multiple transcription factors, including three auxin response factors (i.e. ARF) and two MYBs, can interact with the MaSBE2.3 promoter to regulate its expression [70]. Overall, some research progress has been made on the structural genes of the starch biosynthesis pathway in banana, but the regulatory mechanisms of key genes are still unclear.

## Fruit ripening

Bananas are climacteric fruits and can be harvested at the mature green stage. Then, they are shipped to wholesale markets, where ethylene is applied to artificially ripen them to a golden-yellow colour with optimal consumption quality [81]. Postharvest ripening of banana fruit is a genetically programmed, highly coordinated, and irreversible process regulated by plant hormones, environmental factors, and both transcriptional and post-translational gene modifications. This process ultimately determines key fruit quality traits, including colour, texture, flavour, and aroma [82]. Fruit ripening is a complex process involving multiple gene and transcription factor interactions.

The hormone ethylene is crucial for regulating banana fruit ripening. The fundamental enzymatic processes involved in the synthesis of ethylene are clearly outlined and involve 1-aminocyclopropane-1-carboxylic acid oxidase (ACO), 1-aminocyclopropane-1- carboxylic acid synthase (ACS), and S-adenosyl-l-methionine synthase (SAMS). Both ACO and ACS have been previously identified as limiting enzymes in the ethylene biosynthesis pathway [83–86]. During the postharvest banana-ripening period, the manifestation of *MA-ACS1* and *MA-ACO1* aligns with the observed ethylene production [84]. Among the 10 ACS gene pairs, *MaACS7*/*MbACS7* shows high expression levels during fruit ripening and is predominantly expressed in the B-genome. Similarly, out of the nine ACO gene pairs, three (*MaACO2*/*MbACO6*, *MaACO3*/*MbACO7*, and *MaACO8*/*MbACO13*) are highly expressed during fruit ripening, showing dominant expression in the B subgenome. The increased ethylene production and accelerated ripening of ‘Fenjiao’ (FJ) fruits compared to ‘Baxi’ may be attributed to the principal manifestation of ethylene biosynthesis and genes associated with ripening processes, along with the augmentation of the ACO gene cluster within the B genome [17].

The process of ethylene signaling holds a paramount importance in the control of banana fruit maturation [87]. MaAP2a has been regarded as a key transcriptional inhibitor with strong inhibitory activity by repressing the expression of 15 starch degradation-related genes by binding to the GCC-box or AT-rich motif in their respective promoters [88]. The role of EIN3 in blocking ethylene production has been demonstrated by motif deletion. EIN3-NAC and MADS-type positive feedback loops have been used to control fruit ripening [13]. MaERF012 functions as a transcriptional activator, controlling fruit ripening and triggering the expression of genes responsible for chlorophyll degradation, starch metabolism, and cell wall breakdown [89]. A comprehensive analysis has identified fifteen ERF gene members, denoted as *MaERF1* through *MaERF15*, in the context of banana fruit biology. During ripening or upon ethylene stimulation, *MaERF9* exhibits upregulation, while *MaERF11* displays downregulation, specifically in the peel and pulp. MaERF11 exerts inhibitory control over the promoters of *MaACS1* and *MaACO1*, thereby dampening their functional output. Conversely, MaERF9 is found to enhance the promoter activity of *MaACO1* [90]. MaERF9 interacts with the Dof transcriptional repressor, MaDof23, in banana fruits to regulate ripening-related genes [91]. Seventeen EIL genes were isolated, but no further function identification has been performed [87, 92]. Recently, 13 *MaEIL* genes were isolated, among which *MaEIL9* has been identified as a key factor in the ripening process of tomatoes [93].

Other hormones, such as ABA and auxin, interact with ethylene to control banana fruit ripening. The cooperative transcription regulation by ethylene F-box protein (EBF1) and ABI5-like protein in managing chilling-induced softening of FJ bananas has been elucidated [94]. Auxin-responsive MaIAA17-like protein interacts with the ethylene-insensitive 3-binding F-box protein (MaEBF1), thereby facilitating the upregulation of *MaNOL*, *MaBAM8*, *MaPL8*, and *MaSUR14*. MaCCCH33-like2 interacts with ABI5-like and MaEBF1, resulting in the enhanced binding and activation of promoters of genes related to starch and cell wall degradation [95]. MaIAA17-like exerts its influence on fruit maturation by modulating the expression of genes associated with chlorophyll degradation (*MaNOL* and *MaSGR1*), starch breakdown (*MaBAM6* and *MaBAM8*), and cell wall modification (*MaSUR14* and *MaPL8*), thereby orchestrating the regulatory processes involved. [96]. Brassinazole resistant (BZR) proteins MaBZR1/2 inhibit the transcription of ethylene biosynthetic genes, such as *MaACS1*, *MaACO13*, and *MaACO14*, suggesting their negative roles in facilitating the optimal maturation process of bananas [97].

Starch degradation and cell wall polysaccharide metabolism contribute greatly to banana fruit ripening [16, 98–100]. The breakdown of starch is facilitated by enzymes including α-amylases (AMY), β-amylases (BAM), isoamylase (ISA), and starch phosphorylase (DPE) [17, 71, 81]. Cellulose, hemicelluloses, polygalacturonase (PG), pectin lyase (PL), pectin methylesterase (PME), beta-D-xylosidase (XYL), xyloglucan endotransglycosylase/endohydrolase (XTH), expansins (EXP), beta-galactosidases (Gal), mannanase (MAN), lectin (Lec), glycoside hydrolases (GHs), carbohydrate esterases (CEs), and beta-glucosidase (GLU) are enzymes capable of degrading cell walls [100–102]. The identification of genes linked to enzymes responsible for starch and cell wall breakdown has been accomplished [100, 103]. With regards to starch degradation, the AMY and BMY gene families have a significant size expansion in both the A and B genomes in contrast of other plant genomes. Six highly expressed genes—MbAMY-2, MbAMY-3, MbAMY-8, MbBMY-6, MbBMY-8, and MbDPE-2—are associated with the B subgenome in FJ, likely contribute to the acceleration of the fruit ripening process by enhancing starch degradation [17]. A total of 38 genes implicated in starch degradation, with direct relevance to fruit maturation and softening processes, have been successfully pinpointed [81, 104].

The regulation of ethylene biosynthesis at the transcriptional level during banana fruit ripening has prompted scientists to investigate this further [17, 84, 87, 90, 105] (Table 2). Xu *et al.* [105] identified 287 distinct, differentially expressed cDNAs during the initial stages of banana fruit ripening, which were triggered by ethylene biosynthesis. Among the specific banana genes, transcription factors (TFs) from the MYB and AP2/ERF families—typically associated with regulating fruit ripening—were identified [16]. Multiomics analysis during banana fruit ripening identified differentially expressed genes and accumulated proteins and metabolites in the peel, with key transcription factors belonging mainly to the ERF and bHLH families [116]. Kuang *et al.* [117] performed temporal gene regulatory network analyses, identifying 25 TFs, such as *MaMADS32*, *MaNAC78*/*MaATAF*, and *MaSPL1,* as key candidates in modulating various ripening-related pathways. MaMADS-box transcription factors are crucial for banana fruit ripening, primarily by regulating ethylene biosynthesis, which in turn controls key ripening processes such as softening, colour change, and enzyme production [15, 66, 67, 111, 118]. MaMADS36 interacts with MaOFP1 (an ovate family protein) and regulates fruit ripening by controlling key carbohydrate and cell wall metabolism genes [118]. Another study shows that MaMADS1 is involved in a regulatory cascade with MaNAC083 and MaACS1/MaACOs to control ethylene biosynthesis [119]. Other TFs like R2R3-MYB TF MaMYB, MaBEL1, and the zinc finger protein MaCCCH33-Like2, play a pivotal role in regulating banana fruit maturation by facilitating the activation of genes crucial for cell wall and starch hydrolysis [95, 99, 103].

**Table 2:** TFs and target genes in banana for fruit ripening

Family	TFs	Targets genes	Regulation	Reference
bZIP	MabZIP93	MaPL2, MaPE1, MaXTH23, MaXGT1	up	[106]
	MabZIP21	MaACS1, MaACO1	up	[107]
BZR/BES	MaBZR1/2	MaEXP2, MaPL2, MaXET5	down	[86]
AP2/ERF	MaDEAR1	MaPME3, MaPG1, MaPL, MaEXP1/3, MaXTH10	down	[99]
	Ma AP2a	MaGWD1, MaPWD1, MaSEX4, MaLSF1, MaBAM1-MaBAM3, MaAMY2B/2C/3A/3C, MaMEX1/2, MapGlcT2 - 1 / 2 - 2	down	[88]
	MaERF9	MaACO1	up	[90]
	MaERF11	MaEXP2/7/8, MaACO1, MaACS1	down	[108]
	MaERF012	MaGWD1, MaAMY3, MaPL8, MaEXP-A8, MaXYL23-like	up	[89]
BEL	MaBEL1	MaAMY3, MaXYL32, MaEXP-A8	up	[103]
BSD	MaBSD1	MaEXP1/2	up	[109]
Dof	MaDof23	MaEXP1/2/3/5, MaXET7, MaPG1, MaPME3, MaPL2	up	[91]
LBD	MaLBD1/2/3	MaEXP1/2	up	[109]
TCP	MaTCP5/19/20	MaXTH10/11	up	[103]
bHLH	MabHLH6	MaGWD1, MaLSF2, MaBAM1/2/8/10, MaAMY3/3C, MaSA2/3, MapGlcT2aBA	up	[81]
	MabHLH28	MaGWD1, MaLSF2, MaPG3, MaPE1, MaPL5, MaPL8, MaEXP1, MaEXP2, MaEXPA2, MaEXPA15	up	[110]
EIL	MaEIL2	MaAMY3, MaISA2/3	up	[93]
	MaEIL9	MaAMY3D, MaBAM1	up	[93]
MADS	MaMADS36	MaGWD1, MaBAM9b, MaACS7	up	[14, 85]
	MaMADS1/2	no	up	[111]
	MA-MADS5	MASPS, MAACS1, MAACO1, MAExp, MALec	up	[112]
MYB	MaMYB3	MaGWD1, MaSEX4, MaBAM7/8, MaAMY2B/3/3A/3C, MaMEX1, MapGlcT2-1, MabHLH6	down	[99]
	MaMYB16L	MaISA2, MaLSF1, MaMEX2, MaBAM1/2/4/7/8, MaAMY3/3C	down	[113]
WRKY	MaWRKY49	MaPL	up	[114]
	MaWRKY111	MaGWD1, MaPG3, MaPE1, MaPL5, MaPL8, MaEXP1, MaEXP2, MaEXPA2, MaEXPA15, MaLSF2, MaACO1, MaACS1	up	[110]
Cys-Cys-Cys-His(CCCH)	MaCCCH33-like2	MaISA2, MaSUR14-lik**e,** MaXYL23	up	[95]
NAC	MaNAC1 MaNAC-like (MaNAP4/5)	MaCESA7, MaCESA6B no	up up	[115] [22]

Post-transcriptional modifications, such as histone deacetylation, ubiquitination, and phosphorylation, also play an important role. MaERF11-MaHDA1-MaACO1, MaMYB4-MaBRG2/3, MaXB3-MaNAC19-MaSPS1, MuMADS1-MaUBA, MaBAH1-MaMYB60, MaMPK2-MabZIP93, and MaMPK6-3/11-4-MabZIP21 regulatory modules have been identified, enriching the banana fruit ripening theory [106–108, 114, 119, 120].

Bananas are hypersensitive to high (> 24°C) or low (below 11°C) temperatures, and abnormal temperatures cause cellular membrane damage, flavour loss, and abnormal fruit ripening [104, 121–124]. Cold stress significantly reduces the transcript and protein levels of EBF1, ABI5-like, and fruit softening-related genes, resulting in disruptions to fruit softening and ripening in FJ bananas [94]. Banana WRKYs are strongly induced by cold, mediating enhancer-promoter interactions that regulate key browning pathways, such as cold tolerance [93]. Yang *et al.* [125] revealed an MYB TF-mediated regulatory network after ripening banana fruit at low temperature. High-temperature stress leads to stay-green ripening and speeds up firmness loss and peel senescence in bananas, primarily by influencing hormone signaling and energy pathways as well as stress defence mechanisms [123]. Wei *et al.* [119] identified a transcriptional regulatory network, named MaBAH1-MaMYB60-CCG, that responds to high temperatures, regulating chlorophyll breakdown and green ripening in bananas. Luo *et al.* [126] established a post-translational regulatory network of MaNIP1-MaNYC1, which maintains chlorophyll and causes green ripening in bananas under high temperatures. In addition to protein-level regulation, miRNAs also play a crucial role in cold stress responses. Forty-two miRNAs have been identified as differentially expressed under cold and heat stress. miR393-TIR1/AFB phasiRNA production is uniquely enriched under cold stress conditions [122].

## Fruit quality

Starch is a key constituent of banana fruit, typically accounting for 20–25% of the fresh weight at the time of harvest [69, 70]. Starch synthesis and breakdown occur concurrently [127]. The rapid starch degradation and conversion to soluble sugars directly influences fruit texture, flavour, and shelf life [14, 128], which are comprehensively regulated by target genes, TFs, and other factors. Many enzymes and encoded genes, including *MaGWD1*, *MaLSF2*, *MaBAM1*, *MaBAM2, MaBAM3c* (*MaBMY6*), *MaBAM9b* (*MaBMY7*)*, MaBAM8, MaBAM10*, *MaDPE*, *MaAMY3*, *MaAMY3C*, *MaISA2*, *MaISA3, MapGlcT2-2, MaSPS, MaSS, MaNI,* and *MaAI*, participate in banana fruit starch degradation and sugar accumulation [17, 76, 99, 129, 130]. TFs, such as MabHLH6, MaWRKY111, MaEBF1, MaNAC67, and MADS-box, play key roles in regulating starch degradation [14, 81, 99, 103, 110, 113].

Bananas are a rich source of carbohydrates, potassium, dietary fibre, proteins, vitamins C and E, polyunsaturated fatty acids, carotenoids, flavonoids, phytosterols, gallocatechin, catechin, and other polyphenols, contributing to a healthy diet with beneficial effects on overall human health [131]. The chemical composition of pro-vitamin A in bananas, particularly through carotenoids like beta-carotene, makes them an intriguing tool for supplementation. [132–138]. The carotenoid composition in banana pulp is specific to the genotype and is linked to variations in glycolysis, amino acids, and the ripening method [20, 139–141]. Phytoene synthase (PSY), carotenoid cleavage dioxygenase 4 (CCD4), and MaLCYε are key regulatory enzymes for carotenoid biosynthesis [137, 142–145]. MaSPL16 modulates carotenoid biosynthesis by activating the transcription of *lycopene β-cyclase* (*LCYB*) genes [146]. MaEIL9 positively regulates the transcription of *MaDXR1*, *MaPDS1*, *MaZDS1*, and *MaSPL16* to elevate carotenoid production induced at high temperatures [93]. *MaPSY* is regulated by MaMADS7 [67]. Seventy-nine differentially expressed TF genes may be responsible for *LCYB* regulation [147].

Anthocyanins are phenolic compounds responsible for plant pigmentation and play crucial roles in various biological functions. They are produced through the phenylpropanoid pathway, catalyzed by structural genes, including anthocyanidin synthase (ANS), chalcone synthase (CHS), chalcone isomerase (CHI), dihydroflavonol 4-reductase (DFR), flavanone 3-hydroxylase (F3H), and flavonoid 3-glucosyl transferase (3-GT). Anthocyanins are crucial in the development of both the purple peel in bananas [148] and the red peel of *Musa* AAA ‘red green’ [149]. Several anthocyanin biosynthesis genes, such as *ANS_1*, *F3’5’H_7*, *F3H_2*, *CHS_9*, *4CL_13*, and *PAL_4*, have higher expression levels in immature pericarps [150]. MaMYB4 contributes to the biosynthesis of anthocyanin as a component of the MYB-bHLH-WD40 (MBW) regulatory complex. It acts as a negative regulator by binding to the promoters of key biosynthesis genes, such as CHS, ANS, DFR, and bHLH, suppressing anthocyanin production [151]. In contrast, MaMYBPAs, when overexpressed alone or with MaMYC, promote proanthocyanidin accumulation and activate the transcription of related biosynthesis genes in banana fruit [152]. Additionally, MaMYBA1, MaMYBA2, and MaMYBPA2 are components of the MBW complex, which, in collaboration with bHLH and WD40 proteins, activates the promoters of anthocyanidin synthase and dihydroflavonol 4-reductase in *Arabidopsis thaliana*, two key enzymes in the anthocyanin biosynthesis pathway [153].

Banana fruit is abundant in flavour and aroma, containing over 250 volatile compounds from various chemical classes, including esters, ketones, terpenes, and aldehydes [154]. The ester group is a significant contributor to aroma [155, 156]. The main volatile compounds, including isoamyl acetate, butanoic acid, 3-methyl-3-methylbutyl ester, hexanal, trans-2-hexenal, and 1-hexanol, fluctuate throughout the different stages of ripening [157]. Compounds like methyl butyrate, 2,3-butanediol diacetate, and α-phellandrene have also been identified in bananas, contributing to their unique aroma and flavour, which are important for consumer appeal and fruit quality [158, 159]. In addition to the various volatile compounds contributing to banana flavour, transcription factors play a crucial role in regulating the biosynthesis of these compounds. For example, MabZIP4 directly binds to the BanAAT promoter, while MabZIP5 binds to the promoters of MaMT1, MaACY1, MaAGT1, and BanAAT through the G-box motif, activating the transcription of key aroma biosynthetic genes [160]. MaNAC029 transcriptionally activates genes associated with aroma compound synthesis by directly modulating their promoter activity during ripening [119]. 1-MCP decreases the production of flavour-contributing volatile esters such as isoamyl isobutyrate, isoamyl acetate, trans-2-hexenal, and hexanal, while significantly increasing hexyl acetate production at the full ripening stage [146, 161].

## Biotic stress

Banana plants are vulnerable to various biotic stressors that cause significant yield losses and reduce fruit quality [162–169]. Specifically, *Fusarium* wilt of banana triggered by the fungus *Fusarium oxysporum* f. sp. *cubense* (*Foc*) race 1 (*Foc* race 1) and *Foc* tropical race4 (*Foc* TR4) has caused significant damage on banana plantations [163]. A virulent form of this pathogen, ‘*Foc* race 1’ is the most widely distributed and severely harmful wilt pathogen of banana [21, 22, 42, 65, 164–166]. Black sigatoka, or black leaf streak (BLS), is another fungal disease that affects banana production. It is caused by *Mycosphaerella fijiensis* Morelet, and symptoms of infection include black spots on the leaves [12, 165, 167]. Bacterial diseases, such as banana Xanthomonas wilt (BXW) caused by *Xanthomonas campestris* pv. *musacearum*, are another threat to banana production [169]. Symptoms of this disease include yellow leaves and premature fruit drops, and it can cause up to 100% yield loss [12]. In addition, banana streak viruses (BSVs), banana bract mosaic virus (BBrMV), and banana bunchy top virus (BBTV) are the most prominent viral pathogens affecting bananas. BBTV (genus *Babuvirus*, family Nanoviridae) is the deadliest banana virus for all banana cultivars [168].

The molecular mechanism of the interaction between banana and *Fusarium* wilt has been increasingly elucidated with the advent of next-generation sequencing technologies [170, 171]. Genes relating to ion flow and jasmonic acid biosynthesis, phenylalanine metabolism, salicylic acid signaling, and long chain noncoding RNA (lncRNA), including *2-oxoglutarate Fe(II) dependent oxygenases* (*2OGD*), *PR*, *lysine motif-containing receptor-like kinases 1* (*MaLYK1*), *LRR-RLP*, *MYB36*, *PAL*, and *Macma4_11_g19760*, are involved in the banana disease resistance process [21, 22, 172–183]. Overexpression of *β–1-3 endoglucanase*, *endochitinase*, *Ced9* (apoptosis suppressor gene), *BAG1* (bcl-2-associated homologe), *RGA2* (resistance gene analog 2), and *MpbHLH* in bananas enhances their resistance to *Fusarium* wilt [2, 20, 184–187]. Transgenic *Arabidopsis* lines overexpressing MaWRKY24 are more sensitive to *Foc* TR4 [188]. Silencing *Velvet*, *FTF1*, and the ergosterol biosynthesis gene can confer effective resistance against harmful pathogens in bananas [188]. Recently, the insertion of repeat sequences in the *Foc* TR4 *RAG2* gene promoter was identified in most diploid and triploid bananas. Meanwhile, it was found that the receptor-like protein (RLP) locus, including *Foc* race 1-resistant genes, is absent in the Gros Michel Ze subgenome [22].

Modern biotechnological breeding techniques, including genetic engineering, have been used to improve the biotic stress of bananas, but most of them have been used for research purposes, not yet commercialized [2]. In 2024, the first genetically modified banana variety (QCAV-4) resistant to *Foc* TR4 through overexpressing the *RGA2* gene was allowed for commercial cultivation by the Office of the Gene Technology Regulator in Australia [189]. Currently, biological control is the main strategy for preventing *Foc* [190]. Antifungal rhizobacteria have received extensive attention [191]. Application of *Trichoderma reesei* isolate CSR-T-3, *Streptomyces* ma. FS-4, *Actinomycete Streptomyces* sp. strain H3-2, *Pseudomonas aeruginosa* Strain 91, lipopeptide-producing *Streptomyces, Serendipita indica*, *Dictyophorae echinovolvata*, *Bacillus amyloliquefaciens* YN0904, *B. subtilis* YN1419, and *Bacillus velezensis* YN1910 has resulted in significant reductions in disease severity [191–198]. Crop rotations with pepper and eggplant have significantly reduced the incidence of banana wilt disease through legacy effects [199].

## Abiotic stress

Banana production is highly sensitive to extreme environmental conditions, including heat, cold, drought, excessive rainfall, salinity, and strong winds, all of which can significantly reduce yields [200]. Notably, there is genotype-specific variability in stress tolerance, with the B-genome imparting enhanced resilience to drought and water stress [201, 202]. WRKY, MYB, NAC, bZIP, MADS-box, bHLH, and ERF have been annotated under various stressors [64, 177, 203–211]. miR395, miR397, miR408, miR535, miR156, miR172, and lncRNAs play a key role in the cold stress response [118, 122]. Some banana aquaporin (*MaAQP*) genes, lignin biosynthesis genes, *MaAGPase*, *MaMPK5*, *MAPKKK*, *MAPKK*, *MaCCO*, *MaU-box*, *LysM*, *SRO*, *lysyl oxidase* (*LOX*), *Expansin*, MKK2 family genes, *MaROP5g*, *MaCCS*, *MaGHMP*, and *WOX* have shown strong activation in response to stress, suggesting they could be key targets for improving the resilience of banana cultivars to abiotic stress factors [46, 91, 177, 183, 197, 198, 205, 212–217]. Overexpression of *MaPSY, MusaDHN-1, MusaWRKY71, MusaNAC042, MusaNAC29, MaPIP2-7*, and *MaDREB1F* in bananas significantly increased abiotic stress tolerance [144, 203, 205–207, 218, 219]. Besides, both the MaMAPK3-MaICE1-MaPODP7 pathway and the PYL-PP2C-SnRK2-mediated ABA signaling pathway can significantly enhance responses to abiotic stress in bananas [46, 220]. Histone deacetylation and ubiquitination participate in the response to cold in early-stage leaves during their seedling phase, when they are more susceptible to environmental stresses [104, 221].

## *In vitro* regeneration and somaclonal variations

**Figure 3:**
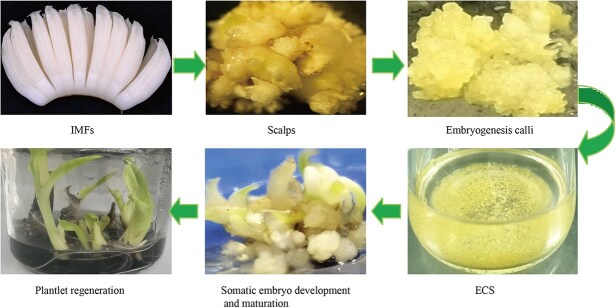
Schematic overview of *in vitro* banana regeneration via embryogenic cell suspension (ECS). Immature male flowers (IMFs) and scalps are the preferred explants for generating renewable ECS cultures in seedless banana cultivars [12, 223]

Establishing a high regeneration system is the major requisite for banana genetic improvement. The banana somatic embryogenesis regeneration system, derived from embryogenic cell suspension (ECS) cultures, serves as an optimal resource for mutation breeding, somatic hybridization, and genetic transformation [222–226]. The preferred explants for establishing ECS are immature male flowers and shoot tip-derived scalps. There are four recognized procedures: embryogenic callus (EC) induction, ECS initiation, somatic embryo development and maturation, and plant regeneration for somatic embryogenesis (Fig. 3) [12, 226]. ECS are not easily obtainable for all banana genotypes, but they have been successfully generated for several key cultivars. These include Cavendish, ‘Gros Michel’, and Plantain varieties such as ‘Agbagba’ and ‘Orishele.’ Additionally, ECS has been produced for other AAB group bananas like ‘Gonja Manjaya’ and ‘Sukali Ndiizi’, as well as some *Musa* ABB varieties, such as ‘Bluggoe’ and ‘Saba’. Certain ECSs have been widely applied in transformation and genome editing to create genetically modified bananas, many of which demonstrate significant resistance to fungal pathogens [12, 223].

**Figure 4:**
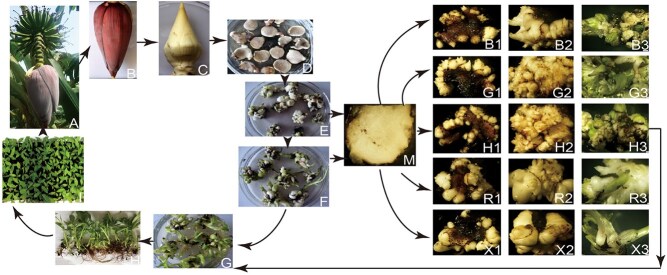
Regeneration process of five Banana genotypes. A, B: floral apex; C: 3-cm long floral apex; D: 1- to 2-mm thick tissue slices; E, F: shoots regenerated from slices; G: regenerated banana roots; H: transplantation; I: plantlets; M: slices taken from E and F; B1-B3: various stages of regeneration for ‘Baxi’ (Cavendish subgroup, AAA); G1-G3: various stages of regeneration for ‘Gongjiao’ (Pisang Mas subgroup, AA); H1-H3: various stages of regeneration for ‘Red banana’ (Red subgroup, AAA); R1-R3: various stages of regeneration for ‘Rose’ (AA); X1-X3: various stages of regeneration for ‘Xinglongnaijiao’ (Pisang Awak subgroup, AAB) [227]

Another vigorous and repeatable regeneration system of direct organogenesis was developed and is suitable for use with five banana varieties of ‘Baxi’ (Cavendish subgroup, AAA), ‘Gongjiao’ (Pisang Mas subgroup, AA), ‘Red banana’ (Red subgroup, AAA), ‘Rose’ (AA), and ‘Xinglongnaijiao’ (Pisang Awak subgroup, ABB) (Fig. 4) [227]. Several genes have been transformed into bananas to improve abiotic resistance and fruit quality [14, 219].

## Genetic improvement and breeding

### Conventional breeding

Hybridization can merge traits from parent plants, such as resistance to pathogens and pests, shorter growth cycles, higher fruit yields, and improved quality characteristics. In bananas, this process was first documented approximately a century ago in Trinidad [228]. New cultivars, such as the FHIA series, NARITA series, ‘Zhongjiao 9’, ‘Fenza 1’, ‘Guangfen 1’, ‘Jinfen 1’, and Nanjiao series, have benefitted from crossbreeding programmes in Brazil and Honduras in the Americas; Cameroon, Ivory Coast, Nigeria, Tanzania, and Uganda in Africa; and China and India in Asia, along with Guadeloupe in the Caribbean [228–231]. The FHIA series included FHIA-17 (AAAA genotype), FHIA-23 (AAAA genotype), FHIA-01 (AAAB genotype), and FHIA-21 (AAAB genotype), which are suitable for fresh or processing, exhibiting different resistance to black leaf spot disease, bunchy top disease, and nematodes [228, 229]. Among twenty-seven NARITA hybrids from East African highland bananas (EAHB) breeding programme at the International Institute of Tropical Agriculture (IITA), only ‘NARITA 7’ was officially released to farmers in Uganda. Although pollination of EAHB can be conducted throughout the year, the seed set and germination is low [230]. ‘Zhongjiao 9’ and ‘Fenza 1’ from the Guangdong Academy of Agricultural Sciences in China exhibited high resistance to Fusarium wilt disease. ‘Guangfen 1’ from the Guangdong Academy of Agricultural Sciences in China showed resistance to banana leaf spot disease, bunchy top disease, black spot disease, and anthracnose disease, but susceptible to Fusarium wilt disease. ‘Jinfen 1’ was selected and bred by the Guangxi Plant Culture Seedling Co., Ltd. in China. It had strong disease resistance and can resist black spot disease, bunchy top disease, and leaf rot disease but it is susceptible to Fusarium wilt disease and root knot nematode. In addition, Nanjiao series from South Subtropical Crop Research Institute, China Academy of Tropical Agricultural Sciences, have the characteristics of resistance to disease and drought stress [228–231]. However, ‘Goldfinger (FHIA-01)’, ‘Guangfen 1’, and ‘Jinfen 1’ are among the few improved varieties that have industrialized applications. The genetic restrictions of sterility and parthenocarpy make it difficult and time-consuming to obtain an excellent variety with polymorphic traits. Therefore, modernizing conventional breeding has become an important requirement for efficiency in the proposing new varieties. Extensive efforts are underway to characterize genetic diversity in breeding programs [232] and to implement genomic selection to accelerate the breeding process and to increase genetic gains [233–235].

### Mutation breeding

Mutation breeding, particularly through radiation mutagenesis, has been used to develop new banana cultivars by inducing random genetic changes in vegetatively propagated bananas. It was used to rapidly fix desirable traits, such as disease resistance [236]. Over 2000 mutant varieties in crops have been officially registered, with significant economic impact globally, and a few of them were developed in bananas [237, 238]. Several export cultivars are derived from somatic mutants of the Cavendish cultivars [239, 240]. However, radiation mutagenesis introduces a high number of untargeted mutations, making the process less controlled and often leading to unintended effects.

### Transgenics and gene editing

Banana breeding could significantly benefit from more targeted approaches that enable precise genetic improvements while preserving the plant's overall genetic identity. Some of the advancements in this area are further outlined below.

#### Fruit quality improvement

Precise genetic modification and genome editing have been widely used to develop improved banana varieties. Fruit ripening and quality are always targets for genetic improvement. Suppression of two MADS-box TFs, namely MaMADS1 and MaMADS2, in ‘Grande Naine’ by anti-sense or RNA interference exhibits specific ripening delay and extended shelf-life phenotypes by affecting ethylene and respiration [111]. Overexpression or antisense expression of *MaMADS36* in red banana significantly improved or suppressed ethylene production, fruit softening, and the conversion of fruit starch to soluble sugars [14]. *MaACO1*-edited Baxi fruit exhibited reduced ethylene synthesis and extended shelf life [241]. Great efforts have been made to develop genetically modified bananas with enhanced vitamin A and iron contents [242]. The Fe and Zn contents increased 6.32 and 4.58 times, respectively, in the leaves of ferritin (from soybean) transgenic Cavendish banana [243]. *MusaFer1* overexpression increased the iron content and anti-oxidative stress ability of Cavendish banana [244]. *Psy2a*, *PSY1*, and *CRTL* genes have been used to develop transgenic banana plants rich in pro-vitamin A [134]. *MtPSY2a* overexpression in Cavendish banana resulted in a pro-vitamin A content of 55 mg/g, which was 20–50-fold higher than that of the control [134]. Most Cavendish varieties have low levels of beta-carotene in their flesh. Editing the fifth exon of the banana *LCY ε* gene resulted in lines with a six-fold increase in fruit beta-carotene content [137]. Targeting *RAS-CCD4* in Rathali resulted in beta-carotene accumulation in the roots of the mutant lines [145]. Editing the *MaGA20ox2* genes in ‘Gros Michel’ resulted in semidwarf mutants [44]. Editing *RAS-PDS1* and *RAS-PDS2* in Rasthali by CRISPR/Cas9 resulted in mutant lines with decreased chlorophyll and total carotenoid contents [231].

#### Biotic improvement

The development of transgenic bananas to enhance their resistance to fungal pathogens has been widely reported. However, most of the transformed broad-spectrum resistance genes are nonplant or nonbanana. For example, antibacterial peptide (magainin) analogues from *Xenopus laevis MSI-99* have been used to enhance the resistance of transgenic Rasthali to FOC race 1 and black leaf streak [245]. Similarly, stacking the Trichoderma harzianum endochitinase gene with grape stilbene synthase in transgenic bananas conferred complete resistance to fungal infection [246]. These transgenic events were further tested through four-year field trials, and several banana lines showed tolerance to BLS. Overexpressing the rice chitinase gene in transgenic banana Gros Michel exhibited resistance to black Sigatoka disease [247]. *Human Lysozyme* from *Homo sapiens* [248], anti-apoptosis gene *Bcl-xl*, *Bcl-2* from *H. sapiens* and Ced-9 from *Caenorhabditis elegans* [185, 249], *Ace-AMP1* from *Allium cepa* [222], *ferredoxin-like protein* (*Pflp*) from *Capsicum annum* [189], *Sm-AMP-D1* from *Stellaria media* and sweet pepper [250, 251], *Thchit42* from *Trichoderma harzianum* [186], *PhDef1* and *PhDef2* from Petunia [252], *Thaumatin-Like Protein* and *PR-5* gene from rice [252], and *β-1,3-glucanase* from soybean [181] have shown significant resistance against FOC races 1 and 4. Resistance to *Fusarium* wilt in bananas has been enhanced by utilizing banana-derived genes associated with disease resistance. For example, overexpressing the *RGA2* or *Ced9* gene in transgenic Cavendish bananas enhances resistance against FOC TR4 under confined field trials, offering a potential solution to combat this devastating disease [2]. Recently, these TR4-resistant transgenic Cavendish bananas, known as QCAV-4, have been sent to regulators for approval to be released for commercial use in Australia [253]. Additionally, RNAi silencing of the targeted genes of FOC race 1 has shown significant resistance in transformation events [252]. Silencing velvet, ftf1, *MusaDAD1*, *Musa BAG1*, *MusaBI, MpbHLH, MaLYK1*, and ERG6/11 increased resistance to FOC races 1 and 4 [20, 177, 187, 190, 252]. Silencing *REP* or expressing small interfering RNAs targeted against the viral replication initiation gene confers banana BBTV resistance [254, 255]. Research to increase resistance to BXW in transgenic bananas has also been reported [12, 255, 256]. Transgenic banana plants expressing sweet pepper *Hrap* and *Pflp* genes have shown complete resistance against *X. campestris* pv. *musacearum* in field trials [12, 255].

#### Abiotic improvement

Transgenic technology and gene editing technology have been increasingly used for improving banana stress resistance. Overexpression of *MusaWRKY71*, *MusabZIP53*, *MusaDHN-1, MusaSAP1, AhSIPR10*, *AhcAPX*, *MpMYBS3* l, *MusaNAC68, MusaNAC042,* and *MusaNAC68* leads to altered abiotic stress responses [48, 183, 203, 205, 219, 257–259]. Aquaporin-encoding gene AQPS is involved in plant tolerance to abiotic stressors, such as drought, high salinity, and low temperature. Overexpression of *MusaPIP1;2, MusaPIP2;6, MaPIP2-7, MaSIP2-1*, *and MaPIP1;1* in bananas significantly increased the tolerance of transgenic bananas to drought, salt, and low-temperature stress [221, 260–262]. Although researchers have made efforts to develop a variety of resistant transgenic bananas. So far, there have been no reports of commercialization of these varieties. These studies are based on the assessment of stress resistance under laboratory or greenhouse conditions, and further research is needed before field trials and commercial release.

## Conclusion and prospects

There remains a significant gap in the development of banana varieties that combine desirable traits such as high yield, superior quality, stress resistance, wide adaptability, and good processing characteristics to meet consumer and industry demands. Since 2012, advancements in banana genome sequencing have provided valuable insights into the genetic makeup of various genotypes, paving the way for new research opportunities. First, understanding the domestication and diversification processes that shaped current banana cultivars remains critical. This knowledge can inform breeding programs by clarifying the ancestral background of cultivars, offering valuable insights into reproduce while enhancing key characteristics present in existing diversity. Second, while progress has been made, the functional genes governing important traits, such as yield, quality, and stress tolerance, are still insufficiently understood. This limits the effective application of molecular breeding technologies needed to address these challenges.

Future research should focus on elucidating the molecular mechanisms behind these traits and integrating multiomics approaches to link molecular patterns with agronomic performance. Additionally, establishing efficient, genotype-independent regeneration systems and advancing gene-editing technologies will facilitate the development of new cultivars with enhanced quality, yield, stress resistance, and postharvest traits. Such progress will contribute to more sustainable, resilient banana cultivation practices, addressing the growing demands of global agriculture.
